# Presence of esterase and laccase in *Bacillus subtilis* facilitates biodegradation and detoxification of cypermethrin

**DOI:** 10.1038/s41598-018-31082-5

**Published:** 2018-08-24

**Authors:** Saurabh Gangola, Anita Sharma, Pankaj Bhatt, Priyanka Khati, Parul Chaudhary

**Affiliations:** 0000 0001 0708 4444 grid.440691.e G.B. Pant University of Agriculture and Technology, College of Basic Science and Humanities, Department of Microbiology, Pantnagar, 263145 India

## Abstract

Ubiquitous presence of cypermethrin as a contaminant in surface stream and soil necessitates to develop potential bioremediation methods to degrade and eliminate this pollutant from the environment. A cypermethrin utilizing bacterial strain (MIC, 450  ppm) was isolated from the soil of pesticide contaminated agriculture field and characterized by using polyphasic approach. On molecular basis bacterial isolate showed 98% homology with *Bacillus subtilis* strain 1D. Under optimized growth conditions, bacteria showed 95% degradation of cypermethrin after 15 days and the end products of cypermethrin biodegradation under aerobic conditions were cyclododecylamine, phenol, 3-(2,2-dichloroethenyl 2,2-dimethyl cyclopropane carboxylate,1-decanol,chloroacetic acid, acetic acid, cyclopentan palmitoleic acid, and decanoic acid. Amplification of esterase (700 bp) and laccase (1200 bp) genes was confirmed by PCR which showed a possible role of these enzymes in biodegradation of cypermethrin. In the presence of cypermethrin Km value(s) of both the enzymes was low than the control. A nobel cypermethrin degradation pathway followed by *B*. *subtilis* was proposed on the basis of characterization of biodegraded products of cypermethrin using GC-MS. Cypermethrin biodegradation ability of *Bacillus subtilis* strain 1D without producing any toxic end product reveals the potential of this organism in cleaning of pesticide contaminated soil and water.

## Introduction

Cypermethrin belongs to a group of synthetic pyrethroid insecticides which are analogous of naturally occurring pyrethrins of botanical origin. It is widely used in agriculture, forestry, Horticulture, public health and house holds for the protection of textiles and to check pest infestation^1–4^. Cypermethrin is used against pests in cotton and vegetable crops as a replacement of organophosphorus pesticide^1^. Pyrethroid insecticides are also used to combat malaria and other mosquito-borne diseases^5^ and constitute common ingredients of household insecticides and control products of ectoparasites of companion animals^6^. Environmental fate of cypermethrin has been studied extensively by various authors. Half-life of cypermethrin in soil varies from 4 to 65 days.

Cypermethrin is an environment pollutant because of its widespread use, toxicity and persistence which may lead to serious damage to non-target organisms and various ecosystems^7^. Therefore, it is necessary to develop a rapid and efficient process to eliminate or minimize the concentration of this pesticide in the environment. Variety of physical and chemical methods are available to treat the contaminants with hazardous chemicals in the soil/ ground water but most of the methods do not actually destroy the hazardous compounds rather help them in binding to the matrix or convert them from one phase to another^8,9^. Biological treatment of chemically contaminated soil is simple, ecofriendly and economic and involves the transformation of complex or simple chemical compounds into non-hazardous forms^10^. For biodegradation, target pesticide acts as a sole source of carbon and energy for the growth of microorganisms which utilize toxic compounds by producing desired enzymes. The specificity of these enzymes involving xenobiotic compounds differs from one microorganism to another. Several microbes involved in biodegradation of β-cypermethrin have been identified in recent years, such as *Serratia* sp. JCN13^11^, *Ochrobactrum lupini* DG-S-01 (Chen *et al*., 2011), and *Pseudomonas aeruginosa* CH7^2^. Three genes, i.e., Estp, pytH, and PytZ, encoding pyrethroid-degrading hydrolases from *Klebsiella* sp. ZD112, *Sphingobium* sp. JZ-1, and *Ochrobactrum anthropi* YZ-1, respectively have been identified by various authors^12–14^.

Use of pesticide-degrading microbial systems for the removal of pollutants from the contaminated systems requires the understanding of ecological, physiological, and biochemical mechanisms of the degrading organisms. Objective of the present study was to isolate and characterize cypermethrin degrading bacterial isolates from of an agricultural field soil. This study may also be exploited in bioremediation and cleaning practices of pesticide contaminated soil, water using the best isolates or gene/enzymes. The present work is very significant in order to degrade the xenobiotic or toxic chemicals into nontoxic end product within a short period of time via ecofriendly and economically.

## Conclusion

*Bacillus subtilis* strain 1D isolated in the present study almost completely metabolized cypermethrin in15 days under laboratory conditions. This is the first report to show the involvement of laccase enzyme in the cypermethrin biodegradation. Metabolism of cypermethrin a bacterial same strain is of vital importance because cypermethrin possess antimicrobial activities hence it prevents the beneficial microflora of the soil. The bacterial isolate harbours the metabolic pathway for the detoxification of the cypermethrin and it completely degrades cypermethrin without leaving any persistent or toxic metabolite. The strain utilizes cypermethrin as a sole source of carbon for growth, which suggests adaptation of *B*. *subtilis* to oligotrophic environment. The ability of the organism to survive at higher concentration of cypermethrin with enhanced degradation potential makes this isolate an ideal candidate for its application in cypermethrin biodegradation.

## Results

### Isolation and characterization of cypermethrin degrading bacterial isolates

**Figure 1:**
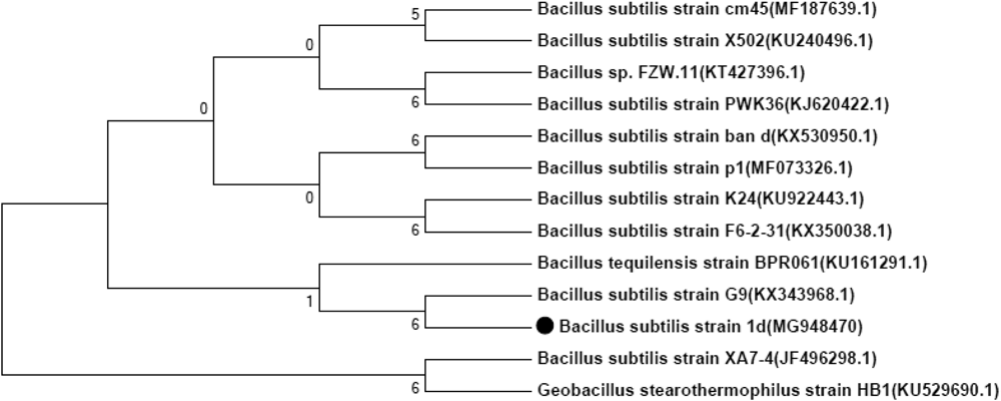
Phylogenetic tree of isolated *Bacillus subtilis* strain 1D constructed using software MEGA 7. The strain showed maximum homology with the *Bacillus subtilis*.

After five rounds of transfer, a sum of 10 bacterial isolates able to grow on cypermethrin as a sole carbon source were recovered from the pesticide contaminated soil of an agriculture field using enrichment culture technique. Recovered bacterial isolates were grown with cypermethrin upto 500  ppm to check their maximum tolerance level for cypermethrin. Out of 10 isolates only one bacterial strain (1D) was able to grow at 450  ppm of cypermethrin. On the basis of above results bacterial strain 1D was selected for further study. Bacterial isolate 1D was aerobic, gram positive, pink in color and had rod shaped cells. The isolate was characterized on the basis of biochemical, physiological and molecular chaacters. Phylogenetic analysis of the 16 s rDNA gene sequences revealed that strain 1D could be grouped among *Bacillus* species as it showed 99% homology with *Bacillus subtilis* (Fig. 1).

### Response surface methodology for cypermethrin degradation by strain 1D

Box-behnken design, based on central composite rotatable design (CCRD) was employed to investigate interactive effect of significant variables like (inoculum size (A), cypermethrin concentration (B), and RPM (C)) on cypermethrin degradation by strain 1D. The experimental design and the response of dependent variables for cypermethrin are described in methods section. Data was processed by response surface regression procedure of Design Expert version-11, software, and results were obtained by fitting with the quadratic model equation (Table 1).

1 $${\rm{Response}}\,({\rm{Y}})=+\,86.98-0.0400{\rm{A}}+4.25{\rm{B}}+2.74{\rm{C}}-4.28{\rm{AB}}-6.88{\rm{AC}}-4.70{\rm{BC}}+4.68{{\rm{A}}}^{2}-8.69{{\rm{B}}}^{2}-6.34{{\rm{C}}}^{2}$$

where (Y) is predicted as % biodegradation of cypermethrin by strain 1D; A, B, and C are the coded values for RPM, concentration and inoculum size respectively.

**Table 1:** ANOVA for the fitted quadratic model for cypermethrin biodegradation.

Source	SS	DF	Mean Square	F-value	p-value
**Model**	1132.93	9	125.88	4.97	0.0231
A-A	1.28	1	1.28	0.0505	0.8286
B-B	145.35	1	145.35	5.74	0.0478
C-C	59.95	1	59.95	2.37	0.1678
AB	73.10	1	73.10	2.89	0.1332
AC	189.06	1	189.06	7.46	0.0293
BC	88.36	1	88.36	3.49	0.1040
A²	92.42	1	92.42	3.65	0.0978
B²	317.96	1	317.96	12.55	0.0094
C²	169.24	1	169.24	6.68	0.0362
**Residual**	177.33	7	25.33		
Lack of Fit	138.80	3	46.27	4.80	0.0818
Pure Error	38.53	4	9.63		
**Cor Total**	1310.26	16			

DF- degrees of freedom, SS- sum of squares.

^*^P level less than 0.05 indicates that the model terms are significant.

R1 = %Biodegradation of cypermethrin (response optimized by RSM).

Figure (S5) shows interaction of one factor with others individually. The factors are speed, pesticide concentration, and inoculum size. From the Fig. S5 it is clear that among the three combinations, optimum percent degradation was 95%. Centre point lies in the parallel to their optimum which, indicates as optimum range of cypermethrin degradation, that is 10 ml(inoculum), 120(rpm) and160  ppm (concentration).

**Figure 2:**
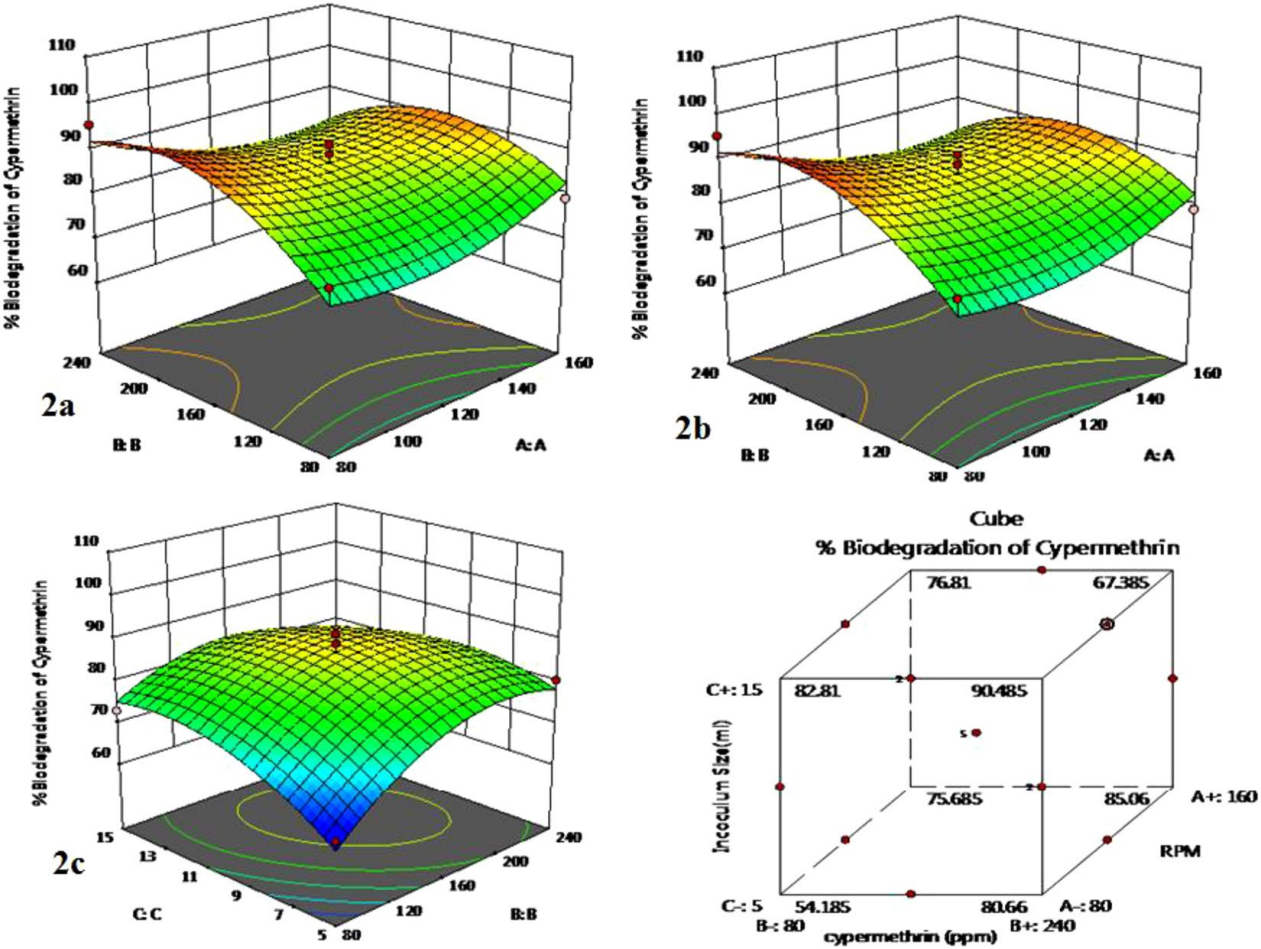
Three Dimensional Graph with multiple factor interaction. Three factor interaction using cube (d). Where A,B and C represents inoculum size (mL), pesticide concentration (ppm) and RPM (shaking speed) respectively.

A three dimensional (3D) response surface graph was plotted to display the effect of pesticide concentration, and speed(rpm) while keeping the value of inoculum size constant (Fig. 2a) and the effect of inoculum size, and speed(rpm) while keeping the pesticide concentration constant (Fig. 2b) and the effect of inoculum size and concentration while keeping the value of speed constant (Fig. 2c). The model predicted that maximum cypermethrin degradation occurred at the stationary point. So the optimum condition for cypermethrin degradation was: speed (120 rpm), concentration (160  ppm) and the inoculum size 10 mL. Multiple factor interaction analysis was also done with the cube which showed same results (Fig. 2d).

### Analysis of cypermethrin biodegradation

**Figure 3:**
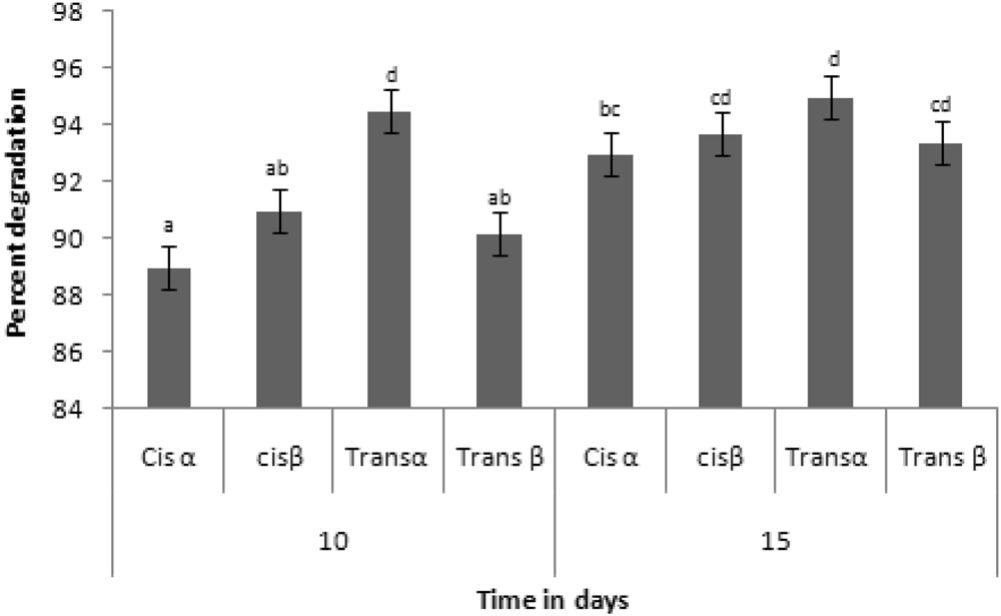
Percent biodegradation of cypermethrin after 10th and 15th days of incubation.

Standard cypermethrin (20  ppm) showed four peaks at different retention time (16.135, 17.00, 17.400 and 17.62 min) representing cisα, cisβ, transα and transβ isomers of cypermethrin respectively in GCanalysis (Fig. S1). The peak area of standard cypermethrin was considered as 100 percent. Degradation of cypermethrin occured in the presence of *Bacillus subtilis* srain 1D. Out of four isomers of cypermethrin (cisα, β and Trans α, β), maximum degradation was observed for trans α followed by cisβ, trans β and cis α. For standard cypermethrin (20  ppm) different peak areas were 1016(cis α), 1208(cis β) and 780(trans α) and 979(trans β). Succsessive decrease in the peak area was observed in cypermethrin isomers after 10^th^ (cisα-116.4, cisβ-124, transα-48, transβ-108) and 15^th^ day (cisα-73, cisβ-86, transα-44, transβ-73) (Fig. S2). Percent cypermethrin degradation was calculated using reduction in the peak area which was 89% (cisα), 91%(cisβ), 94.5%(transα), 90.2% (transβ) after 10 days and 93% (cisα), 93.7%(cisβ), 95%(transα), 93.4% (transβ) after 15 days (Fig. 3 and S3).

### Analysis of intermediates of cypermethrin biodegradation after 15^th^ day of incubation

**Figure 4:**
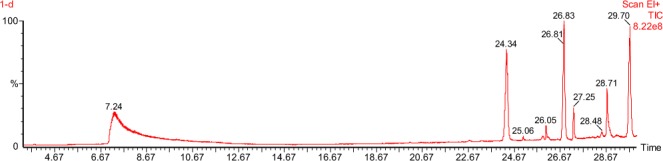
Intermediate metabolites of cypermethrin biodegradation by 1D after 15 days.

GCMS study of biodegraded cypermethrin was conducted at JNU, Delhi, India. Peaks of different compounds were identified on the basis of their mass spectra and library identification program. Peak at retention time of 16.620 min corresponded to cypermethrin standard (control) (Fig. 1). This peak disappeared concomitantly with the formation of another peak with a change in retention time (Fig. 4), which reveals that some new compounds were formed subsequently (Table 2). These compounds were non toxic, as confirmed by literature and library.

**Table 2:** Biodegraded products of cypermethrin after 15 days of bacterial growth in minimal broth.

S.No.	Name of intermediates	Retention Time
1	BENZONITRILE,4-(2-METHYL-1,3-OXATHIOLAN, PHENOL	7.299
2	3-(2,2-DICHLOROETHENYL 2,2-DIMETHYL CYCLOPROPANE CARBOXYLATE	24.356
3	CHLOROACETIC ACID	25.063
4	1-DECANOL	26.839
5	ACETIC ACID	27.252
6	CYCLODODECYLAMINE	27.252
7	CYCLOPENTANE	28.493
8	PALMITOLEIC ACID, DECANOIC ACID	28.729
9	UNDECYL ESTER	29.709

### Proposed Pathway of cypermethrin degradation in bacterial strain 1D

**Figure 5:**
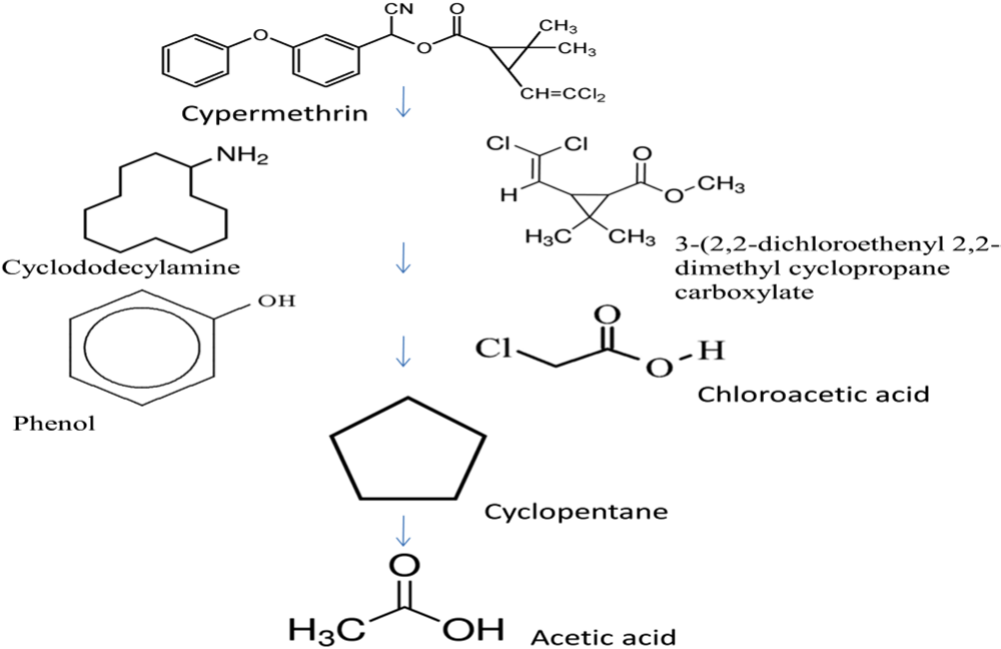
A new proposed pathway of cypermethrin degradation used by bacterial strain 1D.

On the basis of the intermediate products reported after 15 days of incubation of bacterial strain 1D with cypermethrin, a new pathway was proposed for cypermethrin biodegradation (Fig. 5). Hydrolysis of ester linkage of cypermethrin yielded 3-(2, 2-dichloro ethenyl)-2,2-dimethyl-cyclopropanecarboxylate [GC24.356] and cyclododecylamine[GC27.252]. Cyclododecylamine was unstable in the environment and oxidized to form phenol [GC7.299], on the other hand hydrolysis of 3-(2,2-dichloro ethenyl)-2,2-dimethyl-cyclopropanecarboxylate formed chloroacetic acid[GC25.063]. Subsequently phenol reacts with water and forms cyclopentane [GC28.493] which is an unstable compound and transforms into aliphatic compounds like acetic acid [GC28.729] and decanoic acid [28.729].

### Enzyme kinetics

**Figure 6:**
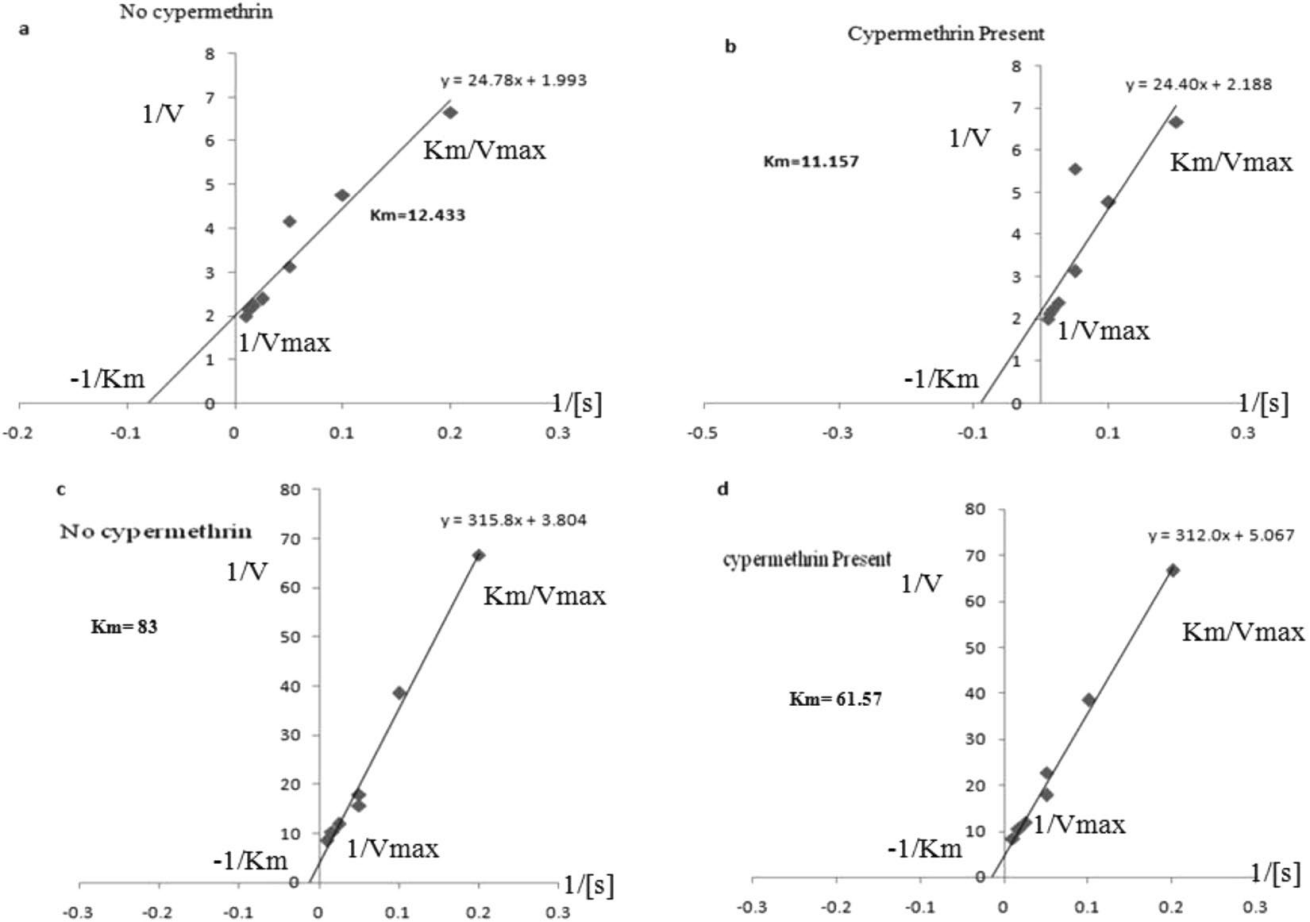
Lineweaver Burk model for esterase and laccase. Figure a & b for esterase and c & d for laccase. In figure a & c absence of cypermethrin, figure b & d presence of cypermethrin. In the presence of cypermethrin km value is decreasing.

The enzyme kinetics of esterase and laccase was analysed in strain 1D by using Lineweaver Burk equation. The concentration of esterase and laccase enzyme was calculated by Lowry’s method. Under normal condition when cypermethrin was not present in the culture medium, concentration of the enzyme in 1D was 92.0 µg/µL while in the presence of cypermethrin (stressed condition) level of esterase increased and was 140 µg/µL. Concentration level of laccase with and without cypermethrin was 62 µg/µL and 42 µg/µL respectively after 15 days. There was a significant difference in the Km values of both the enzymes in the presence /absence of cypermethrin. For esterase, Km values were 11.157 M and 12.433 M in the presence and in the absence of cypermethrin respectively (Fig. 6a,b). For laccase, Km value was 61.57 M and 83 M respectively in the presence and absence of cypermethrin (Fig. 6c,d). These results clearly indicate that under stress conditions competition for enzyme was lower than the normal condition. Vmax values were constant for both the enzymes.

### Amplification of EST and Laccase gene

**Figure 7:**
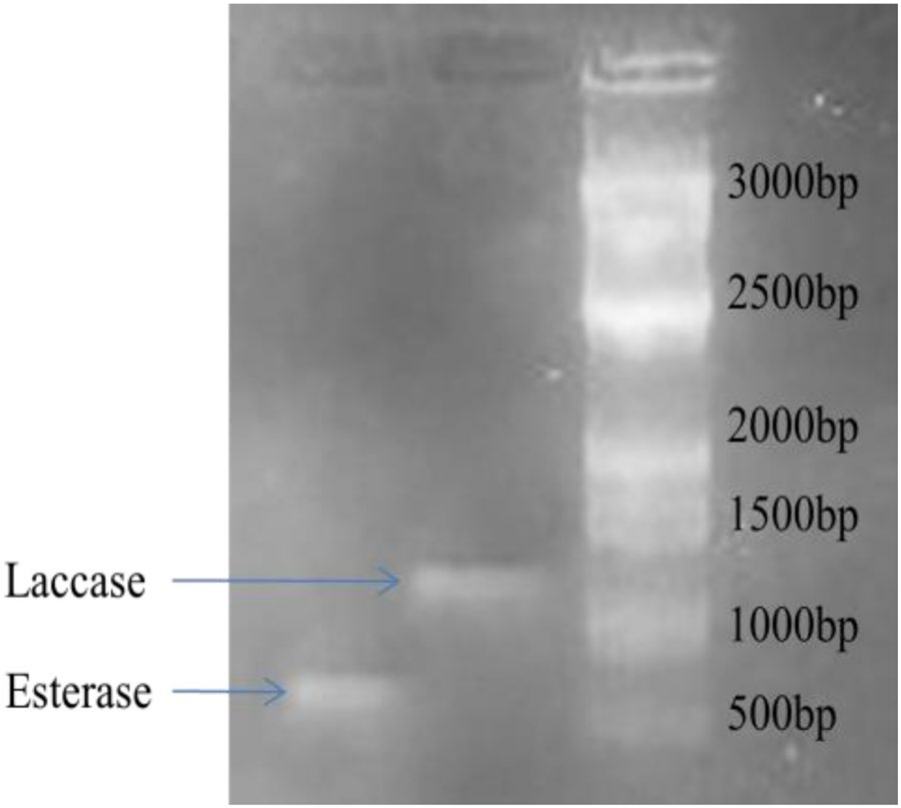
Amplification of Esterase and Laccase genes in *Bacillus subtilis* 1D. Amplificaion of esterase gene product showed bands at 700 bp while laccase amplification was approximately of 1200 bp.

Amplification of EST and laccase genes was observed in strain1D (Fig. 7) Size of the amplicon was approximately 700 and 1200 bp for esterase and laccase respectively. Amplification of laccase and esterase genes indicates that these gene are present in the bacterial genome and their expression is activated in response to the pesticide.

## Discussion

Pyrethroid pesticides are excessively used in agricultural fields to control different pests. Cypermethrin, a member of pyrethroid group of pesticides is invariably used in different crops to get quality yield. Bioremediation using microbial cultures provides a cheap and environmentally friendly way to remove toxic pollutants from the environment. In the present study, a cypermethrin degrading bacterial strain was recovered from pesticide contaminated soil of an agricultural field using enrichment technique and characterized on the basis of biochemical, morphological and molecular (16S rDNA sequencing) characters. The organism showed 99% homology with *Bacillus subtilis* (Fig. 1).

There are some reports of biodegradation of cypermethrin using microbial cultures. Degradation of cypermethrin (100 mgL^−1^) by *Acinetobacter calcoaceticum* MCm5, *Brevibacterium parabrevis* FCm9 and *Sphingomonas* spp RCm6 upto 85% within 10 days^15^. Cypermethrin, endosulfan, imidacloprid degrading bacteria and fungi were also isolated from the rhizospheric fields^16–18^. In order to obtain efficient pesticide degradative bacteria we have screened a wide range of pesticide contaminated soil of agricultural fields, Uttarakhand, India and isolated number of bacterial and fungal isolates which can degrade cypermethrin, fipronil, imidacloprid, chlorpyrifos, carbendazim, melathion, sulfosulfuron, DDT, 2,4D and endosulfan. Few studies have suggested that *Bacillus subtilis* species has potential ability to degrade aromatic compounds (dye) and pesticides like cypermethrin, profenos^19–21^.

*Bacillus subtilis* strain 1D was able to tolerate 450  ppm of cypermethrin and could degrade 240  ppm of the same in minimal medium. This feature gives the advantage of pesticide biodegradation in variable environments, because the organism survived and utilized the toxic compounds when exposed to high concentrations. In a rapid degradation of cypermethrin by *Pseudomonas* spp.and *Bacillus* spp.^4,22^ was observed at 125  ppm with degradation percent of 83%. *Bacillus subtilis* strain 1D could degrade cypermethrin at higher concentration. Possibility of continuous expression of key enzyme(s) responsible for cypermethrin degradation at higher concentration of the contaminant by 1D cannot be ignored. Bacterial isolates engaged in efficient degradation of cypermethrin may be able to grow on the hydrolyzed products of cypermethrin. In present study we presume that metabolic activity of strain 1D was not subjected to complete catabolite repression by high cypermethrin concentration. High cypermethrin tolerance and degradation capability of *Bacillus subtilis* strain 1D, makes this strain suitable for decontamination and remediation of contaminated sites.

Optimum culture conditions for cypermethrin biodegradation by *Bacillus subtilis* Strain 1D were also determined by using response surface methodology (RSM). RSM is an empirical modelling system that has been successfully applied to improve and optimize complex processes, including fermentation for variety of microorganisms^23–25^. Previous studies have shown that application of statistical experimental design in biodegradation processes can result in improved yields of biodegradation with rapid and economical determination of optimum culture conditions using minimal resources^14,26^. In the present study, RSM has been employed to optimize culture conditions which favour cypermethrin degradation. The optimized parameters for cypermethrin biodegradation include speed (rpm), concentration ( ppm) and inoculums size (mL). The results of the experiments were statistically analyzed and the significance and effect of each factor on responses were evaluated (Fig. 2).

*In vitro* percent degradation of cypermethrin using *Bacillus subtilis* in minimal medium was maximum for trans α-(95%) and cis β-(93.7%) after 15 days (Fig. 3). Degradation of trans form of cypermethrin was maximum because this form is less stable than cis. *Bacillus* spp. degraded cypermethrin up to 81.6% within 15 days under standard growth conditions (temperature 32 °C pH 7 and shaking at 116 rpm) in minimal medium^4^. To implement effective biodegradation strategy, it was necessary to analyse the nature of intermediate compounds biodegraded by the bacterial strain. Degradation of pyrethroid insecticides produces more toxic intermediates in biodegradation processes^27^. Our results showed that the strain 1D not only efficiently degraded cypermethrin but also transformed cypermethrin into non toxic forms.

It is presumed that break down of the ester bond of a compound indicates the expression of enzyme carboxylesterases. This enzyme acts as a regulatory enzyme for pyrethroid biodegradation and results in acid and alcohol production^11,21^. Metabolites of cypermethrin have been arranged sequentially to propose a biodegradation pathway in *Bacillus subtilis* (Fig. 5). A new hypothetical degradation pathway of cypermethrin using strain 1D has been proposed because some new intermediates were found during cypermethrin biodegradation (Table 3). GC–MS analysis of biodegraded cypermethrin showed the presence of non toxic metabolites which indicates *Bacillus subtilis* is an effective and suitable strain for the degradation of pesticide belonging to pyrethroid group. To confirm the nature of cypermethrin metabolites, bacterial strain was grown in 15 days old filter sterilized broth containing intermediate compounds of cypermethrin. Surprisingly bacterial strain was able to grow in this broth, which shows the absence of toxic metabolites even after complete degradation of cypermethrin (Fig. 4). However more specific studies are required to test the toxicity of the intermediates of the pesticide.

**Table 3:** The symbols and levels of three independent variables used in Box-behnken design.

Run	A:A	B:B	C:C	% Biodegradation
1	120	160	10	85
2	120	160	10	86.2
3	120	160	10	83.5
4	160	240	10	79
5	120	80	15	73
6	120	240	5	80.3
7	160	160	15	86.5
8	160	80	10	79
9	120	80	5	62.4
10	120	240	15	72.1
11	80	160	5	70.4
12	160	160	5	90.5
13	80	240	10	95.5
14	80	80	10	78.4
15	80	160	15	93.9
16	120	160	10	89
17	120	160	10	91.2

Because of the specificity, esterase and laccase play important role in the degradation of wide variety of pollutants in the environment. Activities of esterase and laccase were elucidated qualitatively and quantitatively for their possible role in cypermethrin biodegradation. Increase in esterase and laccase activity was observed in the presence of cypermethrin as compared to the control. Results indicate induction of esterase and laccase activities in 1D bacterial strain under cypermethrin stressed condition to overcome the stress. Cypermethrin biodegradation was brought about by esterase enzyme which is present in *B*. *subtilis* strain 1D. Role of bacterial esterase has been elucidated for biodegradation of carbamate, organophosphate and cypermethrin. Different forms of pyrethroid hydrolases and their expression have been reported in *Ochrobacterium*, *Bacillus* and *Sphingobium* spp. Esterase belongs to hydrolase group of enzymes and found capable of hydrolyzing a large number of ester bond and ester bond containing compounds^28–30^.

Presence of bacterial laccase is reported in *Azospirillum lipoferum*^31^, *Pseudomonas syringae*^32^, *B*. *subtilis*^33^. Role of fungal laccase is reported in the degradation of chlorpyrifos, liluron and metribuzin^34^. Bacterial laccase is also involved in dye decoloration in *Bacillus subtilis*^35,36^ *Bacillus vallismortis*^37^, *Bacillus subtilis* X1^38^. Very few studies have been reported on biodegradation of pesticide using laccase. Most of the studies were conducted on fungal laccase. Role of laccase in the degradation of cypermethrin was observed in strain 1D. So the presence of these two enzymes in the bacteria makes the organism more prominent candidate for the degradation of wide range of the pollutants.

Minimal medium supplemented with cypermethrin showed low km values which confirms that cypermethrin induces production of esterase and laccase while under normal conditions, km values were high when 1/Vmax is constant. This indicates production of enzyme is under competitive inhibition. This may be because some chemical constituents of the medium may bind to enzyme and act as competitive inhibitors. In the presence of the cypermethrin, laccase and esterase may undergo conformational changes and finally enzymes are free to work which leads to decrease in km value (Fig. 6). The role of esterase in the degradation of indoxacarb has been described which helped in the detoxification of the xenobiotic compounds^39,40^. Thermophillic bacterium *Alicyclobacillus tengchongensis* completely degraded malathion by producing esterase^41^. Presence of laccase gene was also confirmed by gene amplification. The same set of primers used and found amplicons of 600–1500 bp size for laccase^42^. Our results are also relevant to the findings of^42^, as amplicon size of 1200 bp in *B*. *subtilis* was reported. Amplification of esterase gene was observed in strains of *Bacillus* spp. And product was of approximately 550 bp whereas in SA2 it was at approximately 700 bp^3^.

Laccase and esterase genes are major regulatory genes which are responsible for cypermethrin degradation. Esterase is responsible for the formation of alcohol and acid when it reacts with water. Laccase uses molecular oxygen as a co-substrate and converts it into water. The results of EST and laccase amplification were also supported by the result of GCMS where acid and alcohols are formed. Various authors have used fungi, bacteria, plant, animal and microbial enzyme to study the biodegradation of Carbon nanotubes (CNTs), graphene (GRA), and their derivatives along with experimental and molecular simulation methods^43–46^ showed that single-walled carbon nanotube (SWCNT) release would significantly affect the microbial enzyme-catalyzed processes of organic pollutants and lignin model compounds (LMCs) in nature. They found that microbial degradation appears to be the most promising practical application as compared with enzymatic degradation because enzymatic degradation strictly requires a suitable temperature and pH. If environmental conditions are not appropriate, the enzyme activity could be inhibited or disappear. The limitations for microbial degradation are relatively lower because microorganisms can grow under a variety of conditions^47,48^. Composting or addition of compost can simultaneously increase soil organic matter content and soil fertility besides bioremediation (pesticides, hydrocarbon and phenol), and thus it is believed to be one of the most cost-effective methods for soil remediation^47,49^ explored laccase on molecular level for lignin degradation by using molecular docking and molecular dynamics (MD) simulations which provide detailed information about interaction mechanism between laccase and lignin. This is useful to develop new laccases with high lignin-degrading ability in the field of environmental protection and industrial applications.

## Methods

### Chemicals and media

Standard cypermethrin (97% purity) used in this study was obtained from Department of Chemistry of the University. Hexane and other chemicals/reagents used in the study were of analytical-grade and available commercially. Stock solution of cypermethrin (1 mg/ml) was prepared in hexane and stored in dark bottles at 20 °C after filter sterilization.

Nutrient agar containing (gL^−1^) peptic digest of animal tissue 5.0; Sodium chloride 5.0; Beef extract 1.5; Yeast extract 1.5; Agar 15.0; and mineral salt medium (MSM) containing (gL^−1^) (NH_4_)_2_SO_4_, 2.0; MgSO_4_.7H_2_O, 0.2; CaCl_2_.2H_2_O, 0.01; FeSO_4_.7H_2_O, 0.001, Na_2_HPO_4_.12H_2_O, 1.5; and KH_2_PO_4_, 1.5 were used for the isolation of bacterial strains.

### Enrichment and isolation of cypermethrin-degrading Bacteria

Pesticide contaminated soil samples were collected from the agricultural fields of Udham Singh Nagar, Uttarakhand, India. Enrichment and isolation of pesticide degrading bacterial strains was carried out in MSM (minimal Salt Medium) by using enrichment culture technique^17,18,50^. Bacterial colonies with different morphologies appeared on the plates were picked, purified and preserved on nutrient agar in refrigerator. One bacterial isolate showing maximum tolerance for cypermethrin was selected for further study.

### Identification and Characterization of strain 1D

Cypermethrin-degrading bacterial isolate 1D was grown on Nutrient agar at 33 °C for 24 h and identified on the basis of morphological, biochemical, and molecular characters. Genomic DNA of the bacterial strain was extracted^51^. 16S rDNA gene was amplified using universal primers (27 f;5′AGAGTTTGATCMTGGCTCAG3′ and1492r:5′TACGGYTACCTTGTTACGACTT-3′). Amplified PCR product was run on agarose gel and sequenced by Biotech Centre, South Campus,Delhi University. Resulting 16S rDNA gene sequences were compared using BLAST program and phylogeny of the organism was deduced by MEGA 7.0 software^52^.

### Inoculum preparation

To test cypermethrin biodegradation under laboratory condition bacterial inoculum was prepared by growing the isolate in 50 mL Nutrient broth for 24 h at 33 °C under shaking condition at 120 rpm. After incubation, bacterial culture (2 mL) was transferred to MSM (50 mL) containing 20  ppm cypermethrin under aseptic conditions and allowed to grow for 20 days at 33 °C with shaking at 120 rpm. Samples were withdrawn after 10th and 15th day of incubation and residual pesticide was quantified by GC after extraction^17^.

### Optimization of growth conditions of strain 1D for cypermethrin biodegradation

Box-behnken design was explored to optimize the degradation conditions of cypermethrin using bacterial strain 1D. Box-behnken design consisting of 17 experimental runs with three replicates at the centre point was used to optimize the independent variables which significantly influenced cypermethrin biodegradation by *Bacillus subtilis* strain 1D. Three critical factors and their optimal ranges selected in this experiment for the analysis of cypermethrin biodegradation were; inoculum size (5, 10 and 15 mL), cypermethrin concentration (80,160 and 240 ppm) and shaking speed (80, 120 and 160) rpm. Experiment was conducted in minimal medium for 15 days (Table 3).

### Chemical analysis

#### Extraction of cypermethrin from MSM

Supernatant was collected by centrifugation after 10^th^, and 15^th^ day of bacterial growth in the presence of cypermethrin in minimal salt medium, Un-inoculated medium served as control. Two milliliter of MSM sample was centrifuged at 10,000 rpm for 10 min. Supernatant (1 ml) was transferred to Buchner funnel and mixed with sodium sulphate (1 g) and hexane (1 ml). After formation of two separate layers in separating funnel, bottom layer was discarded and upper layer was collected in a round bottom flask and evaporated completely in an evaporator at room temperature. To the left out of the pesticide, 2 ml hexane was added and mixed properly. After filtration, extracted solution was collected in the eppendorf tube and analyzed by GC.

Degradation products of cypermethrin in MSM containing 20  ppm of cypermethrin were determined by gas chromatography- mass spectrometry (GC-MS) equipped with auto-sampler, an on-column, split/ split less capillary injection system, and HP-5MS capillary column (30.0 m × 250 µm × 0.25 µm) with array detection from 30–500 nm (total scan). The operating conditions were as follows: the column was held at 80 °C for 5 min, ramped at 8 °C.min^−1^ to 200 °C (first ramp), held at 200 °C for 5 min, ramped at 15 °C.min^−1^ to 260 °C (second ramp), and then held at 260 °C for 5 min. The temperatures corresponding to transfer line and the ion trap were 280 °C and 230 °C, respectively, and the ionization energy was 70 eV. The injection volume was 1.0 mL with a split ratio of 1:7 at 260 °C. Helium was used as a carrier gas at a flow rate of 1.0 mL min^−1^. The metabolic products of cypermethrin were matched with authentic standard compounds on the basis of mass spectrum by using library database.

### Estimation of esterase and laccase enzyme in bacterial strain 1D

#### Laccase

Tryptone yeast extract medium (0.2% yeast extract and 0.2% tryptone, pH, 7.2) inoculated with 1.0% of 12–14 h old bacterial inoculum was incubated at 37 °C, 150 rpm for 120 h. After incubation, bacterial culture was centrifuged at 6,000 × *g* for 20 min at 4 °C to obtain pellets. Obtained bacterial pellets were washed with phosphate buffer (0.1 M; pH 6.5) containing 10 mM of phenylmethylsulfonyl fluoride (PMSF) to check protease activity in the supernatant before sonication (5 times, 45 s each time with 30 s between each sonication, 20 MHz). The cell extract obtained by centrifugation (14,000 × *g*) at 4 °C for 20 min was used as a source of crude intracellular laccase enzyme. In principle oxidation of guaiacol by laccase results into reddish brown color which is used to measure enzyme activity at 465 nm. Enzyme assay was performed^53^. A blank with 1 ml buffer instead of enzyme acted as control. One activity enzyme unit (U) was defined as the amount of enzyme that oxidizes 1 μmol of guaiacol per min at 25 °C and the activity is expressed in U/L.

#### Esterase

One mL bacterial culture as grown for laccase was transferred to eppendorf tube and centrifuged at 8000 rpm for 10 min (4 °C). Obtained pellets were suspended in 100 mM potassium phosphate buffer (pH 7.5) and centrifuged at 8000 rpm for 15 min at 4 °C. Esterase activity was determined spectrophotometrically at 450 nm according to^40^. The rate of hydrolysis was expressed as micromoles of α- naphthol produced per minute at room temperature and the specific activity of the enzyme is expressed as micromoles of α-naphthol produced per minute per milligram protein at room temperature.

### Kinetics of esterase and laccase by lineweaver burk model

The ability of strain 1D to degrade cypermethrin was investigated in the presence of cypermethrin by Lineweaver Burk quation for esterase and laccase.

2 $${\rm{Equation}}\,{\rm{is}}\,1/{\rm{V}}=({\rm{Km}}/{\rm{V}}\,\max )1/[{\rm{S}}]+1/{\rm{V}}\,\max $$

### Amplification of esterase and laccase genes

To study biodegradation of cypermethrin, major pesticide degrading genes (EST and laccase) were targeted in the test bacterial isolate. Two sets of primers for est amplification were ESTf-5′ATTATACCCGCCCAGTCGCT and ESTr-ATGAATATGCTCCGCCCCGAC3′, and for laccase CulAF-5′ACMWCBGTYCAYTGGCAYGG3′ and Cu4R-5′TGCTCVAGBAKRTGGCAGTG-3′.

For EST, 25 µl of reaction mixture contained: dNTPs mix 1 µl (10 mM), Forward Primers 1 µl (10 pm/µl), Reverse Primers 1 µl (10 pm/µl), Assay buffer 2.5 µl (10X) with MgCl2, Taq DNA polymerase 0.5 µl (3.0 U/µl), Template DNA 4 µl (50 ng/µl). For laccase, reaction mixture contained: dNTPs mix 0.5 µl (10 mM), Forward Primers 2.5 µl (20 pm/µl), Reverse Primers 2.5 µl (20 pm/µl), Assay buffer 2.5 µl (10x) with MgCl2, BSA 3(10 mg/Ml), Taq DNA polymerase 0.333 µl (3.0 U/µl) and Template DNA 5 µl (50 ng/µl). Conditions for PCR reactions were maintained with minor modifications^54^. For EST-initial denaturation (95 °C for 5 min), denaturation (94 °C for 1 min),annealing at (49 °C for 1 min), from step second, 35 cycle repeat, extension (72 °C for 1 min),final extension at 72 °C for 7 min were programmed.For laccase- initial denaturation (94 °C for 3 min), denaturation at (94 °C at 30 sec), annealing (50 °C for 30 sec), from step second 35 cycle repeat, extension (72 °C for 1 min) and final extension at 72 °C for 5 min were maintained.

### Data analysis

Statistical analysis of the data was performed with the help of SPSS. Results were analysed by ANOVA and statistical analysis was performed on three replicates of the data obtained from each treatment. The significance (P < 0.05) of differences was treated statistically by one way ANOVA and evaluated by Duncan test.

## Electronic supplementary material

Supplementary Dataset
