# Supplementary material for: Presence of esterase and laccase in Bacillus subtilis facilitates biodegradation and detoxification of cypermethrin
Source: Sci Rep. 2018 Aug 24;8:12755. doi: 10.1038/s41598-018-31082-5 (PMC6109164; doi:10.1038/s41598-018-31082-5)

SUPPLIMENYTARY FILE

Title- Presence of esterase and laccase in *Bacillus subtilis* facilitates biodegradation and detoxification of cypermethrin

Saurabh Gangola*, Anita Sharma, Pankaj Bhatt, Priyanka Khati, Parul Chaudhary

*Corresponding author Emails: saindsaurabh@gmail.com

Fig-S1: Standard of cypermethrin (20 ppm)

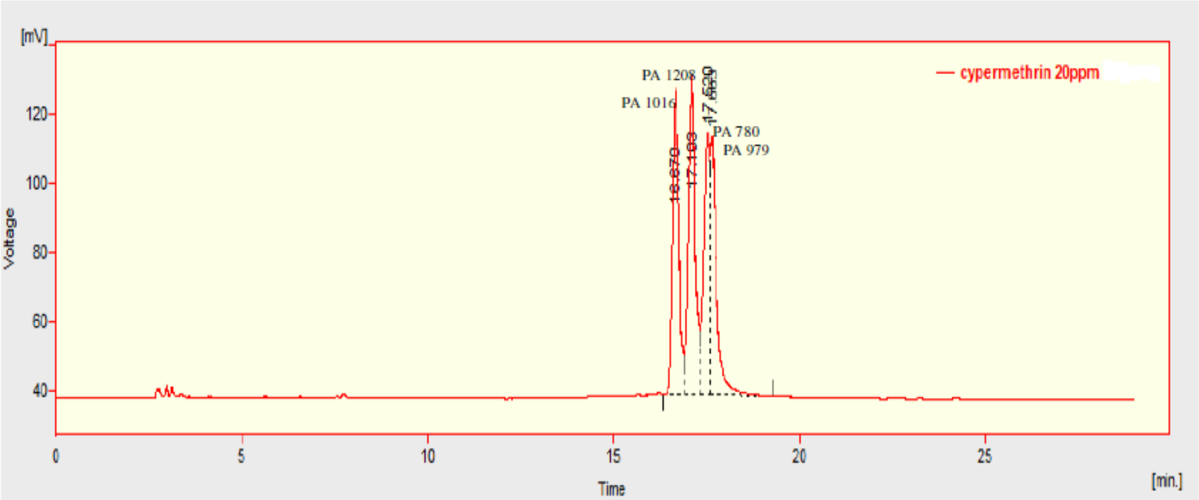

Fig-S2:GC analysis of cypermethrin degradation after 10 day

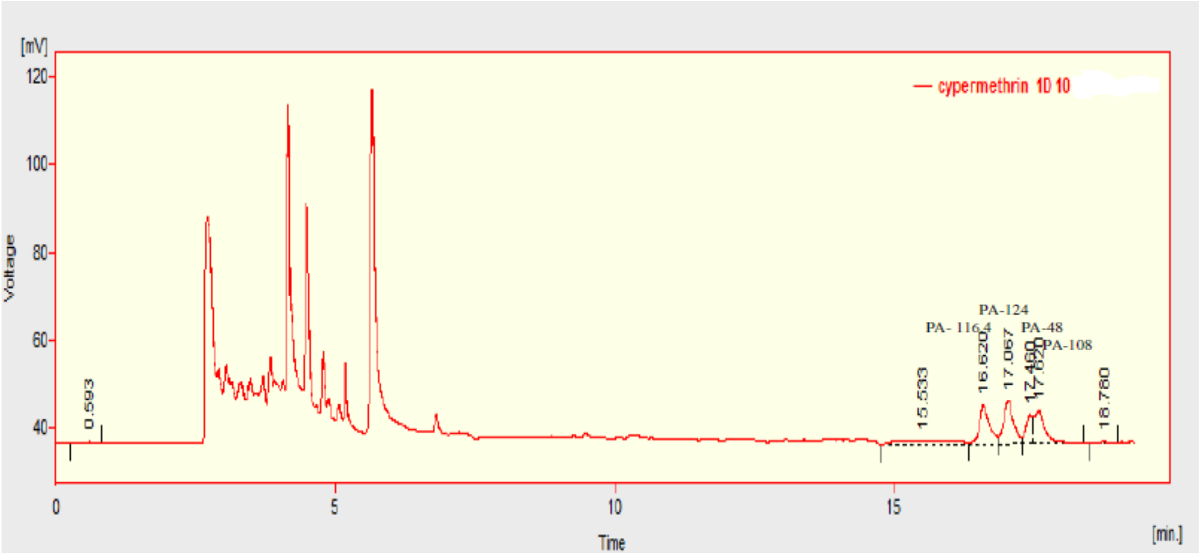

Fig-S3: GC analysis of cypermethrin degradation after 15 day

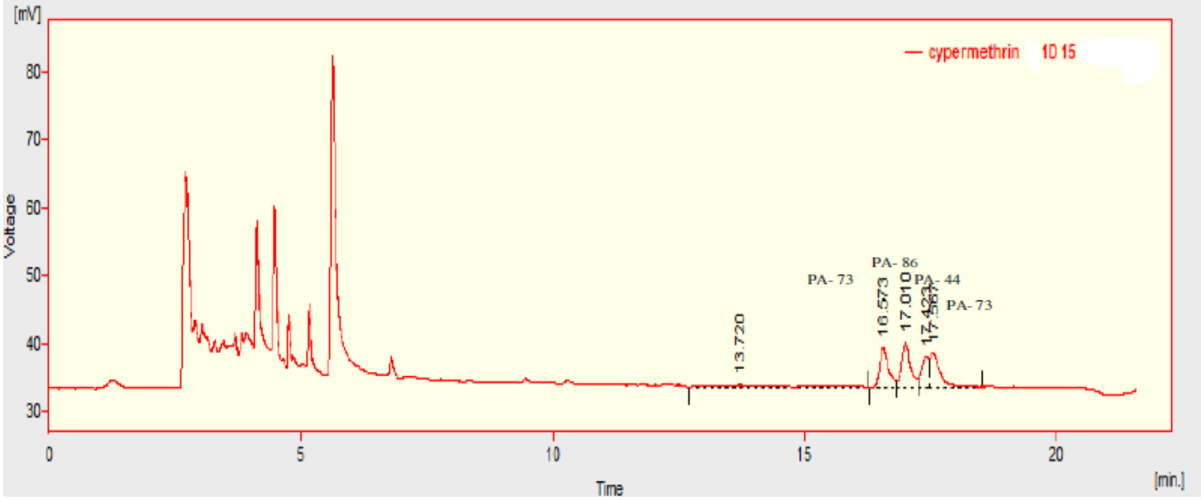

S4: Sequence of isolated strain 1D

CGYWATCWCCRGGCGGCTGGCTCCTAAAGGTTACCTCACCGACTTCGGGTGTTACA
AACTCTCGTGGTGTGACGGGCGGTGTGTACAAGGCCCGGGAACGTATTCACCGCGG
CATGCTGATCCGCGATTACTAGCGATTCCAGCTTCACGCAGTCGAGTTGCAGACTGC
GATCCGAACTGAGAACAGATTTGTGGGATTGGCTTAACCTCGCGGTTTCGCTGCCCT
TTGTTCTGTCCATTGTAGCACGTGTGTAGCCCAGGTCATAAGGGGCATGATGATTTG
ACGTCATCCCCACCTTCCTCCGGTTTGTCACCGGCAGTCACCTTAGAGTGCCCAACT
GAATGCTGGCAACTAAGATCAAGGGTTGCGCTCGTTGCGGGACTTAACCCAACATCT
CACGACACGAGCTGACGACAACCATGCACCACCTGTCACTCTGCCCCCGAAGGGGA
CGTCCTATGTCTAGGACTGTCAGAGGATGTCAAGACCTGGTAAGGTTCTTCGCGTTG
CTTCGAATTAAACCACATGCTCCACCGCTTGTGCGGGCCCCCGTCAATTCCTTTGAG
TTTCAGTCTTGCGACCGTACTCCCCACGGGGAGTGCTTAATGCGTTAGCTGCAGCAC
TAAGGGGCGGGAACCCCCTAACACTTAGCACTCATCGTTTACGGCGTGGACTACCA
GGGTATCTAATCCTGTTCGCTCCCCACGCTTTCGCTCCTCAGCGTCAGTTACAGACCA
GAGAGTCGCTTCGCCACTGGTGTTCTCCACATCTCTACGCATTTCACCGCTACACGT
GGAAKTCCACTCTCCTCTTCTGCACTCAAGTTCCCCAGTTCCAATGACCCTCCCCGGT

TGAGCCGGGGGCTTTCACATCAGACTTAAGAAACCGCCTGCGAGCCCTTTACGCCAT
AATTCCGGACAACGCTTGCCACCTACGTATTACCGCGGCTGCTGGCACGTAGTTAGC
CGTGGCTTTCTGGTTAGGTACCGTCAAGGTACCGCCCTATTCGAACGGTACTTGTTCT
TCCCTAACAACAGAGCTTTACGATCCGAAAACCTTCATCACTCACGCGGCGTTGCTC
CGTCAGACTTTCGTCCATTGCGGAAGATTCCCTACTGCTGCCTCCCGTAGGAGTCTG
GGCCGTGTCTCAGTCCCAGTGTGGCCGATCACCCTCTCAGGTCGGCTACGCATCGTT
GCCTTGGTGAGCCGTTACCTCACCAACTAGCTAATGCGCCGCGGGTCCATCTGTAAG
TGGTAGCCGAAGCCACCTTTTATGTTTGAACCATGCGGTYCAAACAACCATCCGGTA
TTAGCCCCGGTTTCCCGGAGTTATCCCAGTCTTACAGGCAGGTTACCCACGTGTTACT
CACCCGTCCGCCGCTAACATCAGGGAGCAAGCTCCCATCTGTCCGCTCGACTGCASA
AACGWYYCACG

Fig-S5: Effect of one factor on cypermethrin biodegradation

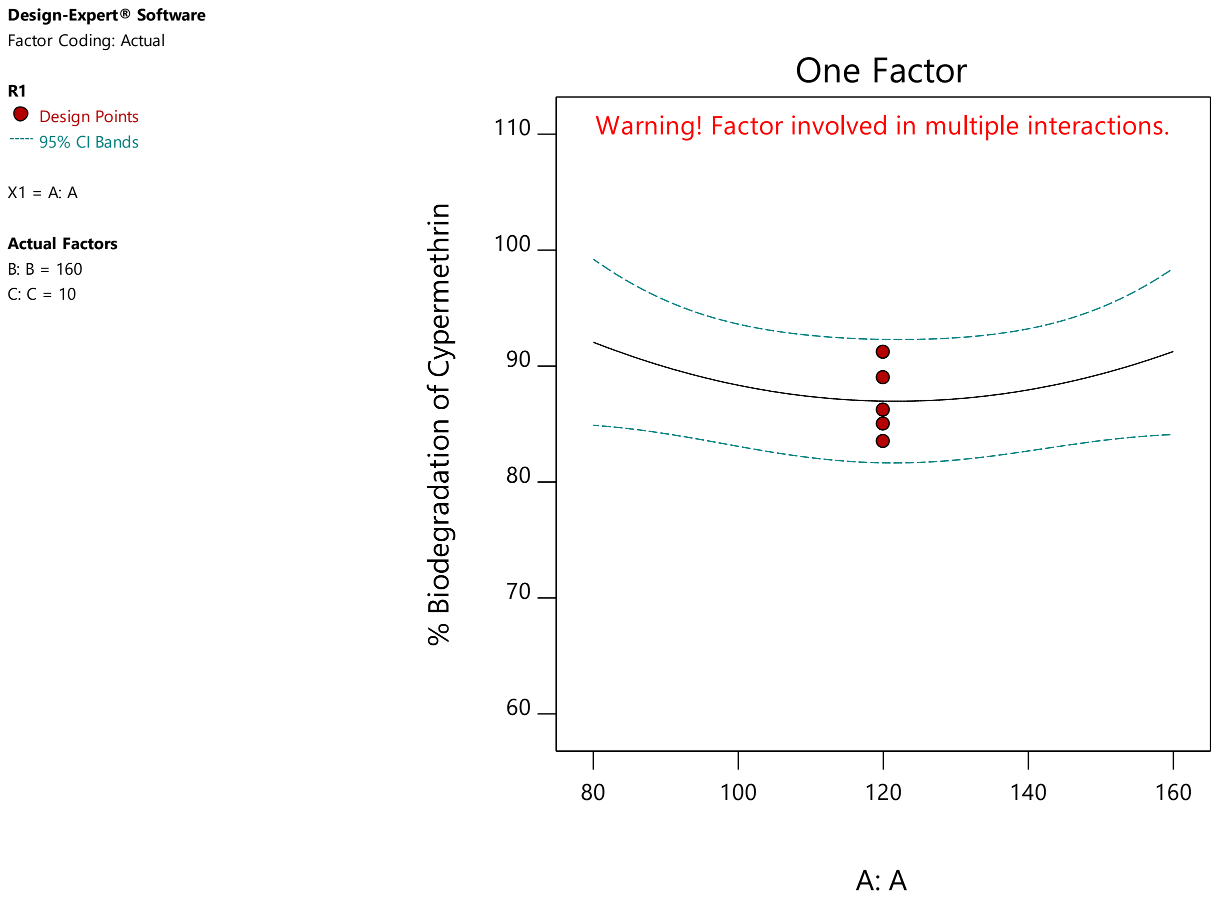

Design-Expert® Software
Factor Coding: Actual

R1
Design Points
95% CI Bands

X1 = B: B

Actual Factors
A: A = 120
C: C = 10

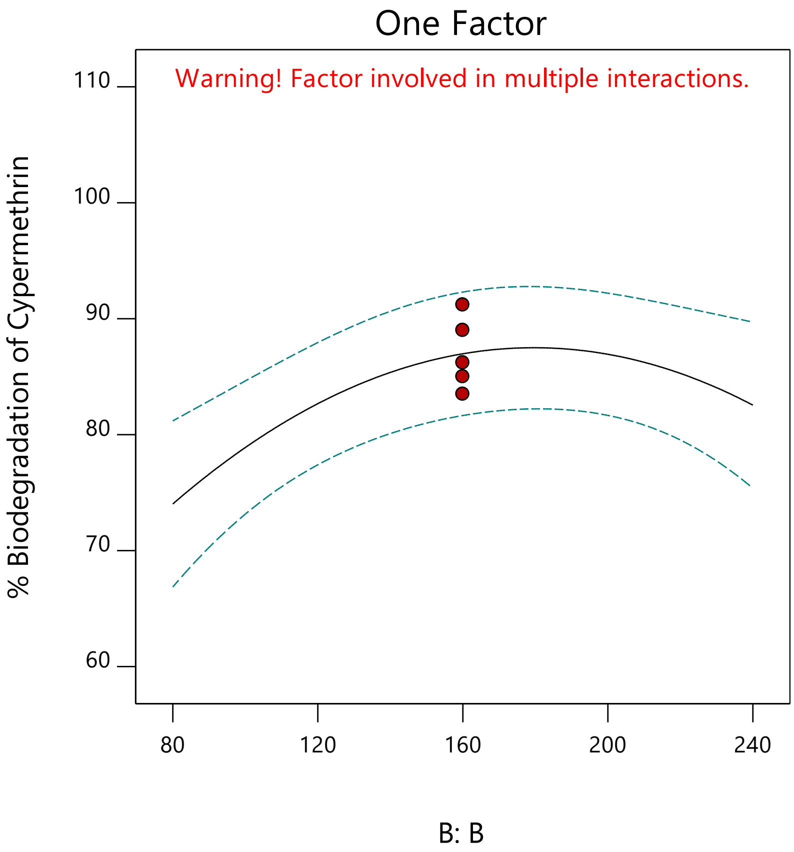

Design-Expert® Software
Factor Coding: Actual

R1
Design Points
95% CI Bands

X1 = C: C

Actual Factors
A: A = 120
B: B = 160

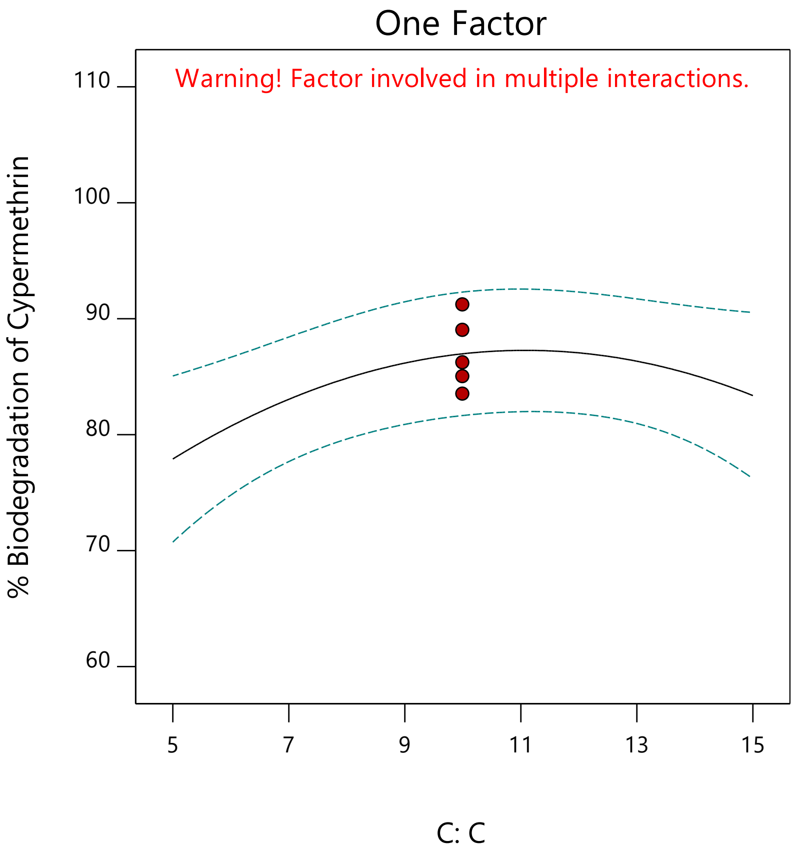

Supplement: Supplementary file 1 — Supplementary Dataset [file 41598_2018_31082_MOESM1_ESM.pdf]
